# Technical innovation to calibrate the gantry angle indicators of linear accelerators

**DOI:** 10.1120/jacmp.v2i1.2630

**Published:** 2001-01-01

**Authors:** Liyun Chang, Sheng‐Yow Ho, Jia‐Ming Wu, Chin‐Yen Yu, Chien‐Chen Sung

**Affiliations:** ^1^ Department of Radiation Oncology Sinlau Christian Hospital Tainan Taiwan Republic of China; ^2^ Department of Radiation Oncology Kaohsiung Chang Gung Memorial Hospital Kaohsiung Taiwan Republic of China

**Keywords:** mechanical quality assurance, good surface, real gantry angle

## Abstract

Finding the actual zero degree of the gantry angle is important in order to perform the mechanical quality assurance (QA) of linear accelerators. To determine real zero, we must locate a “good surface” which could be defined as a plane on the surface of the gantry head that is perpendicular to the direction of radiation. The actual gantry angle could then be defined as the angle between vertical, as indicated by a plumb bob, and the direction of the beam axis that could be indicated by the position of a BB placed in the central axis and its shadow. From this we located the real zero degree and the good surface. The good surface can be applied to check the important mechanical readouts. The technique we introduce could solve the essential problems of a traditional QA technique, as well as taking up an important role in the quality assurance of a patient's treatment.

PACS number(s): 87.56.–v

## INTRODUCTION

In operating a linear accelerator, the gantry rotation readout, collimator rotation readout, gantry rotation isocentricity, and laser alignment are important parameters for commissioning and quality assurance (QA).^1^ ^,^ ^2^ Usually, the physicist will check those items using a level attached to a flat surface beneath the gantry head near the gradicule and rotate the gantry to place the air bubble of the level at the center. However, the surface to which the level is attached is seldom checked. In this paper, we introduce a technique that can be used to calibrate this surface so quality assurance can be achieved.

We use the term “good surface” to identify the surface that the level can be attached to in order to execute mechanical QA. A good surface could be defined in the following way: After we attach the level to the good surface and rotate the gantry to adjust the level's bubble at the center of the horizontal bar, the direction of radiation becomes the same as the direction of gravity. The “real gantry angle” is defined as the angle between the direction of gravity and the direction of radiation. The situation where the direction of radiation is parallel to the direction of gravity would then be defined as the actual zero of gantry rotation.

## MATERIALS AND METHODS

**Figure 1:**
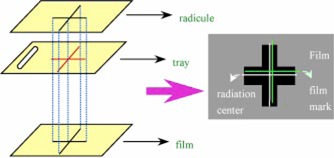
(Color) The setup to find out the radiation center on the tray.

Varian 2100C/D, 2100C, and 600C linear accelerators were used for testing. The collimator rotation isocentricity “star shot” test was performed first to make sure the jaw's center coincided with the radiation center to within specification.^3^ We inserted a tray with a cross mark carved according to the cross hair projection shadow. A film was put on an adjustable plate, which was on the couch and had a surface that could be adjusted to make sure the film was perpendicular to gravity. We also made a cross mark on the film according to the cross hair projection shadow. After closing one pair of jaws and exposing the film, we closed another pair of jaws and exposed the film again, where the collimator jaws were set according to the star shot. When the film was developed, a cross made by the radiation was shown. The center of the cross was compared to the cross hair mark to determine the relative positions of the crosshair and the radiation center. Then the radiation center could be found on the tray (Fig. 1). A small hole was drilled in the tray at the center of radiation. Because the digital gantry angle increases linearly with the mechanical gantry angle within the small angle region, we rotated the gantry to positions with –2°, –1°, 0°, 1°, and 2° to perform the measurement of a real gantry angle and find the real zero degree. A plumb bob was then hung through the drilled hole, and the distance from the hole of the tray to the apex of the bob was set at 50 cm. For marking the film we inked the apex part of the plumb bob. A film was then put on the adjustable plate as described above, and the couch was gently elevated to make the apex part of the plumb gently contact the film. A point mark was made on the film, and the marked position was checked three times by moving the couch up and down. After piercing the mark by gently using a needle, we took away the plumb from the tray and put a BB in the hole. Then we shot the film using the photon beam using 180 monitor units. To prevent scattering, we did not use polystyrene which produces a build up of depth on the film.

**Figure 2:**
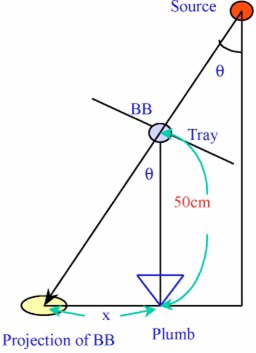
(Color) An illustration of the relationship between the real gantry angle *θ* and *x*(the distance between the projection of the BB and the apex of the plumb bob).

After developing the film, we found a white point (BB shadow in Fig. 2) and a black point (the needle mark). As in Fig. 2, when the distance between the two points is defined as *x*, we can calculate the real gantry angle *θ* by the formula

(1) $$\theta =\tan^{-1}(x/50).$$

The real zero angle could be found by extrapolating the data of real gantry angles to zero, when *x*is equal to 0 and the digital angle is *α*. We rotated the gantry to *α* degrees and reshot the film, trying to make the two points overlap. If they did not, we would rotate the gantry by increments of 0.1° until the two points did overlap, where the gantry digital angle is *α*‘. We then rotated the gantry to digital angle *α*‘, which is the real zero, and attached the level to the surface as suggested by the manufacturer. By shimming under one end of the level, we made the air bubble move to the center. The shimmed surface thus will be the good surface.

## RESULTS

**Figure 3:**
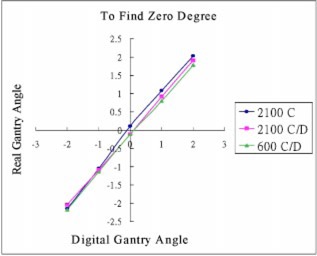
(Color) Results of the real gantry angle measurement of Varian 2100C, 2100C/D, and 600C/D.

Measurements of the real gantry angle using Varian 2100C, 2100C/D, and 600C/D are shown in Table I and Fig. 3. The real angle was obtained from Eq. (1), $\theta =\tan^{-1}(x/50)$, and Fig. 2.

**Table I:** Results of the real gantry angle measurement of Varian 2100C, 2100C/D, and 600C/D.

Gantry angle for testing (digital panel showing)	Varian 2100C Real gantry angle	Varian 2100C/D Real gantry angle	Varian 600C/D Real gantry angle
–2° (178°)	–2.15°±0.06	–2.04°±0.13	–2.18°±0.05
–1° (179°)	–1.05°±0.07	–1.09°±0.10	–1.14°±0.12
0° (180°)	0.11°±0.06	–0.11°±0.00	–0.11°±0.04
1° (181°)	1.08°±0.04	0.92°±0.10	0.80°±0.09
2° (182°)	2.03°±0.03	1.91°±0.07	1.78°±0.08
Air bubble at level center	0.26°±0.03	–0.11°±0.06	0.00°±0.09

After extrapolating the data in Table I to calculate the real zero degree of the gantry and rechecking the position by reshooting the film, we got the digital *α*‘ equal to *α*. When the air bubble was at the center of the level, we set the digital angle of gantry as *β*. The angle between the good surface and the surface suggested by the manufacturer is set at *γ*, which is equal to $\beta -\alpha^{\prime }$, and also equal to the real gantry angle when the air bubble was at the center of the level (Tables I and II).

**Table II:** The list of the digital angle α', where the gantry was at real zero degrees, the digital angle β, where the air bubble was at the center of the level, and the digital angle γ, which is the angle's difference between the good surface and the surface suggested by the manufacturer.

Machine	*α*'	*β*	*γ*
Varian 2100C	–0.1°	0.2°	0.3°
Varian 2100 C/D	0.1°	0°	–0.1°
Varian 600C/D	0.1°	0.1°	0.0°

The data showed that the surface suggested by the manufacturer was around 0.1°−0.3° from the good surface. This value was so small that we could still use the surface suggested by the manufacturer to perform QA. However, the surface suggested by the manufacturer could also become a good surface by shimming under one end of the level. The shims were glued to the gantry and marked for placement of the level. We can then perform the mechanical QA using the good surface to calibrate lasers, the gantry angle, and the collimator angle.

## DISCUSSION AND CONCLUSION

The accuracy of this method is affected by the quality of plumb bob, the diameter of the hole drilled in the tray, and the tray insertion.

(1) Quality of plumb bob. In our tests, the line of the string should pass through the apex part of the plumb bob. This can be tested by taking two photographs from different views. The string line must coincide with the point of the plumb in each photograph. This criterion was met in our tests.

(2) The diameter of the hole drilled on the tray. The center of the hole drilled was the radiation center, and its radius was 1 mm. Therefore, the maximum error would be around $\tan^{-1}(0.1/50)=0.11{}^{\circ}$.

(3) Tray insertion. If the tray does not hold tightly, it could cause an error of 1 mm (i.e., another 0.11°). So, in our tests, the tray was held tightly. Hence, the accuracy in our test would be around 0.1°±0.05°(±0.05° is from the precision of the gantry digital angle, which is used to obtain the real zero degree.)

The precision of our tests was affected by the string position in the drilled hole, the wind from the air conditioner, and the precision of the ruler. The plumb string could lie on the edge of the hole when the gantry was tilted through an angle. Because wind might cause some problem, we needed to take time to wait for the plumb to become steady. The ruler offered a precision of 0.25–0.5 mm.

According to our tests, we could use the surface suggested by the manufacturer to perform the QA because it is within specification for clinical use. With our technique, the gantry angle could be recognized with a precision of around 0.1°. Therefore, we could delicately calibrate gantry rotation readout, collimator rotation readout, gantry isocentricity, and laser alignment.

## ACKNOWLEDGMENTS

The authors would like to thank Dr. John Cunningham for his review of the manuscript.
